# Standardization of the Monocyte Monolayer Assay: Lessons Learned From Laboratory Optimization and Literature Review

**DOI:** 10.1002/jcla.70381

**Published:** 2026-10-20

**Authors:** Shamee Shastry, Kavya Babu, Deepika Chenna, Ganesh Mohan, Kartik Iyer

**Affiliations:** ^1^ Department of Immunohematology and Blood Transfusion Kasturba Medical College, Manipal Academy of Higher Education Manipal India

**Keywords:** antibodies, cellular assay, laboratory standardization, medical diagnostics, quality diagnostics, quality education

## Abstract

**Objectives:**

Several variables influence the outcome of cellular assays. We conducted a comprehensive literature review to identify the factors affecting the Monocyte Monolayer Assay (MMA) and subsequently standardized the assay through systematic in‐house laboratory trials.

**Methods:**

The study was conducted in two phases. Phase 1 involved a literature review using PubMed and Scopus databases to identify factors influencing the assay. Phase 2 included experimental trials to evaluate the effects of these factors. Monocytes were isolated from buffy coat bags, and assay parameters, such as centrifugation speed, monocyte adherence conditions, RBC sensitization, incubation time, and staining, were systematically standardized. A blinded evaluation was employed for slide reading to assess the influence of procedural factors on assay reliability.

**Results:**

A literature review using the PubMed and Scopus databases identified key variables influencing MMA performance. Twelve sequential trials were conducted to identify the effects of these variables on assay outcome. Optimal centrifugation speed and time was 600 × *g* for 15 min, and the use of four buffy coat bags improved monocyte yield to 8.3 × 10^6^/mL. Dilution with Phosphate buffer saline (PBS, pH 7.2) and use of Histopaque provided consistent cell recovery. A 1‐h incubation yielded optimal monocyte adherence with minimal lysis. The final protocol demonstrated high reproducibility and improved visualization of phagocytosis.

**Conclusion:**

This study emphasizes the importance of standardizing the MMA procedure before implementation. Optimizing the parameters to ensure enhanced monocyte yield, reduced cell lysis, and reliable interpretation for assessing the clinical significance of red cell alloantibodies is essential.

## Graphical abstract

Standardization of monocyte monolayer assay in a developing country. This study highlights the significance of literature review and adopting best techniques for standardization of a new procedure at individual center before implementing into practice.

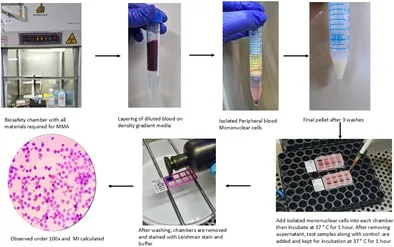

## 1 Introduction

Transfusion support to patients with antibodies to high frequency red cell antigens, autoimmune hemolytic anemia with auto or auto‐ and alloantibodies, and those with antibodies to unknown clinical significance is challenging. The risk of hemolysis and the in vivo survival of the transfused red cells is unpredictable. Both in vivo and in vitro methods are available for predicting the hemolysis and red cell survival [1].

In vivo methods to predict red cell survival include Chromium‐51 (Cr^51^) and biotin‐labeled assays or a biological crossmatch. However, in view of the unknown clinical significance of the red cell antibody, the patient is at risk of developing hemolysis [2]. In vitro methods include antibody‐dependent cellular cytotoxicity (ADCC), the chemiluminescence test and the monocyte monolayer assay (MMA) which help predict the clinical significance before red cell transfusion [3].

Of the in vitro assays, MMA is the most widely used in vitro method for determining the clinical significance of irregular antibodies against red cell antigens, with the first research being published in 1980 using human monocyte macrophages [4, 5]. Though it has been described in the literature for more than 40 years now, it remains a rarely performed test, with only a few centers successfully standardizing it [6]. The primary challenge lies in the numerous variables that influence the assay, making it a complex, multistep procedure that requires careful optimization. These steps are crucial for establishing the credibility of an assay, minimizing errors, and ensuring consistency in clinical decision‐making. Tong et al. investigated assay variables and observed that MMA can be reliably performed on whole blood samples collected in ACD, maintained at room temperature for up to 36 h, provided that physiological pH is preserved throughout the assay [7]. To ensure reliable results, Tran et al. proposed a curated pool of volunteers with proven monocyte reactivity [8]. When a higher yield of monocytes is required to evaluate patient serum against multiple donor units to select the best matched blood unit, careful consideration of assay‐related variables is critical. During efforts to standardize the Monocyte Monolayer Assay (MMA) using an allogeneic cell source, we observed that several pre‐analytical, analytical, and biological factors significantly influenced assay performance and interpretation. Therefore, in the present manuscript, we reviewed the existing literature to identify the key variables reported to affect MMA results for identifying the clinical significance of red cell antibodies in patients with autoimmune hemolytic anemia.

## 2 Materials and Methods

The study was conducted over 6 months (January 2023–June 2023) after approval from the Institutional Ethics Committee. The final protocol was submitted for copyright to the Government of India (Dairy no. LD‐16200/2025‐CO). This study was conducted in two phases.

Phase 1: A structured narrative literature review was performed to identify methodological variations in published monocyte monolayer assay protocols that could influence assay performance and guide laboratory standardization. A simplified search strategy using the keywords “monocyte monolayer assay” was conducted in the PubMed and Scopus databases, with the search restricted to English‐language journal articles published within the last 10 years. Studies outlining different methods for monocyte isolation, RBC preparation, and assay execution were critically analyzed. A comparison of various MMA techniques used in the studies was conducted to identify variations.

Phase 2: Repetitive testing was conducted to assess the impact of key factors, leading to the development of a protocol that yielded the most reliable assay results.

The Monocyte Monolayer Assay (MMA) was performed following these steps: monocyte isolation and control preparation, monocyte monolayer preparation, staining, and interpretation.

Monocyte Isolation: Monocytes were isolated from blood samples collected from buffy coat bags obtained from donors who donated whole blood. Buffy coat bags were obtained following component separation of whole blood collected from voluntary blood donors using triple or quadruple blood collection bags. Monocyte count was assessed by performing differential counts to determine the adequacy of the counts and confirmation of minimal lymphocyte counts. However, due to limited funding, a quantitative purity assessment of monocytes by flow cytometry was not feasible.

Monocyte fixation: A 200 μL aliquot of mononuclear cells (MNCs) was added to each well of the assay plate. The plate was incubated at 37°C for 60 min. Non‐adherent cells such as lymphocytes were carefully aspirated and discarded. The plastic chamber was then carefully removed. The supernatant was aspirated and discarded to ensure that only adherent cells remained for analysis.

### 2.1 Preparation of Positive Control (IgG Coated RBCs)

Serial dilution of IgG anti‐D antiserum (Monoclonal Eryclone^R^ Anti‐D Rho IgG) was prepared using conventional tube technique and the dilution showing 2+ agglutination reaction with Rh D‐positive RBCs in the AHG phase was selected. A pool of 3–5 O Rh D‐positive donor samples was prepared and washed three times with normal saline (3000 RPM for 3 min). A 3%–5% cell suspension was then prepared from the final washed cells. Equal volumes of cell suspension and diluted anti‐D (IgG) were mixed, incubated at 37°C, and washed to obtain IgG‐coated red cells.

### 2.2 Negative Control Preparation

A 3%–5% suspension of red blood cells from a healthy donor was prepared and mixed with PBS. For quality verification, a Direct Antiglobulin Test was performed and confirmed to be negative.

### 2.3 Test Samples

Samples used for MMA testing included serum from patients with autoimmune hemolytic anemia with the presence of antibodies in the serum showing pan reactivity and evidence of hemolysis in vivo. The specificity of the antibodies was characterized to be of Ig G subtype either alone or in combination with Ig M.

### 2.4 Sample Testing

200 μL each of the test sample, positive control, and negative control were added to wells labeled appropriately. The plate was incubated at 37°C for another 60 min. After incubation, the wells were gently rinsed three times with saline to remove unattached cells, and the edges were blotted dry with tissue paper.

### 2.5 Staining and Microscopic Analysis

Two drops of Leishman stain were applied to each chamber for 2 min, followed by 20 drops of PBS buffer on the slide for 8 min. The wells were carefully removed and the slides were washed by dipping them in distilled water. The edges were blotted dry, and coverslips were applied to prepare the slides for observation.

### 2.6 Reading and Interpretation of the Test Results

Once prepared, the slides were observed under a microscope (100×) to count 300 monocytes in each well. The percentage of monocytes showing RBC phagocytosis or adherence was calculated. The cutoff for the assay was 5%, below which it was considered negative, while a value greater than or equal to 5% was considered positive, which was established from previous studies [9, 10].

To minimize bias, a blinding approach was implemented, ensuring that the individual performing the test and the expert validating the results were different. In Addition, unique identification numbers were assigned to the slides for an unbiased assessment.

## 3 Results

### 3.1 Phase 1: Results of Literature Review

The articles were further reviewed to comprehend the various steps involved and their significance in the outcome of the MMA. As per the literature, most of the published articles on the procedure for MMA were modified forms of the initial method described by Gallagher et al. and Branch et al. [11, 12]. The general outline of the procedure includes the isolation of monocytes, adherence of the monocytes to a chamber, addition of sensitized red blood cells to the adhered monocytes, and staining followed by studying the extent of phagocytosis under a microscope.

A comparison of various techniques revealed significant variations in the performance of the MMA including differences in the source of monocyte isolation, temperature conditions, and size of test tubes used for isolation. Additionally, variations were noted in the choice of density gradient media and the type of centrifuge used for both isolation and washing with PBS. Factors such as dilution, culture media, incubation time, and platform used for smear preparation also differed across protocols. Furthermore, discrepancies were observed in the counting methods used to assess assay results, highlighting the need for standardization to ensure consistency and reliability. A comparison of variations in the study protocol from various studies is presented in Table 1 which describes the factors associated with assay performance [3, 7, 13, 14, 15, 16].

**TABLE 1:** Variables identified affecting the procedure of Monocyte monolayer assay from literature review.

Variables	Judd's et al. [13]	Boligan KF et al. [3]	Tong TN et al. [7]	El‐sayed HAN et al. [14]	Merck’s protocols [15]	Kipkeu BJ et al. [16]
Source of Monocyte	15 mL of whole blood from healthy donor. Centrifuged and removed plasma.(150 *g* for 10 min)	Fresh whole blood collected in 2 ACD tubes from healthy donors or patient.	Fresh whole blood collected in 2 ACD tubes from healthy donors or patient.	25 mL of human whole blood were obtained from healthy donor in citrated blood bags	2 mL of defebrinated‐ or anticoagulant treated blood from healthy donors	Whole blood from healthy voluntary donor
Temperature		Store whole blood in ACD tubes at room temperature (18°C to 22°C)	Store whole blood in ACD tubes at room temperature (18°C to 22°C)		At room temperature	
Dilution	The layer formed is dilute with 35 mL of pH 7.3 PBS.	1:1 RPMI 1640 media	Dilute the whole blood 1:1 v/v in warm RPMI 1640 supplemented with 10% fetal bovine serum, 20 mM HEPES, and 0.01 mg/mL gentamicin	Whole blood was diluted 1:1 with RPMI 1640 medium supplemented with 10% fetal bovine albumin and 1% of pencillin/streptomycin	2 mL of defebrinated‐ or anticoagulant‐treated blood and an equal volume of balanced salt solution (Volume 4 mL).	Diluted (1:1) with RPMI 1640 (contains 20 mmol/L HEPES, L‐glutamine, without NaHCO3) plus 10% fetal bovine serum (FBS).
Density gradient media	12 mL of Ficoll‐Paque Plus	15 mL room temperature Ficoll Paque Plus(1:1)	10 mL of blood mixture on top of 3 mL of density gradient‐Ficol Paque	12.5 mL of room temperature Ficoll‐ Hypaque per tube(1:1)	3 mL of Ficoll‐ Paque media and 4 mL diluted blood	Histopaque (15 mL) was dispensed into 50‐mL centrifuge tubes and 15 to 17 mL of the diluted BC
Size of test tube for isolation	50‐mL, conical centrifuge tube.	50‐mL Falcon tubes	15 mL Falcon tube	50 mL conical tubes	10 mL centrifuge tube	50 mL centrifuge tube
Centrifugation for PBSC isolation (speed and time)	800 *g* for 15 min.	700 *g* for 30 min with brakes OFF.	700 *g* for 30 min with brakes OFF	2400 rpm for 20 min (without brakes) at 24°C	400 *g* for 30– 40 min at 18°C to 20°C (brake should be turned off).	700 *g*, RT, 40 min
Centrifuge speed for washing with PBS (speed and time)	400 *g* for 10 min.	350 *g* with full brakes ON for 10 min	350 *g* with full brakes ON for 10 min	1800 rpm for 8 min.	400 to 500 *g* for 10 to 15 min at 18°C to 20°C.	washed with 30 mL of RPMI plus 10% FBS 400 *g*, RT, 7 min
Incubation time	37°C for 60 min.	37°C, 5% CO_2_ for 1 h in a humidified tissue culture incubator	37°C, 5% CO_2_ for 1 h in a humidified tissue culture incubator	37°C, 5% CO_2_ for 1 h in a humidified tissue culture incubator. After adding test samples 2 h incubation		37°C, 5% CO_2_ for 1 h
Platform used for smear	Lab‐Tek tissue culture chamber slides	8‐well chamber slide	8‐well chamber slide	8 welled and 24 welled chamber		22 × 22 mm glass coverslips (Fisher Scientific) treated with poly‐L lysine hydrobromide solution. Coverslips were placed in 35‐mm culture dishes
Counting method	Wright's stain for 3 min and place in Giemsa stain for 20 min	Using a phase contrast microscope and 40× objective lens, quantify phagocytosis using a manual cell counter	Using a phase contrast microscope and 40× objective lens, quantify phagocytosis using a manual cell counter	Air‐dried slides were fixed using 100% methanol and stained using Wright‐ Giemsa or Leishman stain, and counted microscopically by (40×) and oil immersion (100×) lens.		Observed under microscope

### 3.2 Phase II

Following the literature review, we conducted a series of experiments to achieve optimal MMA performance, which required 12 trials to obtain the best results, as shown in Table 2.

**TABLE 2:** Details of the experimental trials performed to optimize the monocyte monolayer assay.

Process	Evaluation	Purpose	Outcome
Monocyte Source Selection (Trials I–III)	Use of buffy bag as monocyte source with varying centrifugation conditions.	To standardize a suitable method for monocyte isolation	Unsuccessful. Reasons being bulging of bags, tube distortion, poor mononuclear layer formation and inadequate monocyte yield (0.25 × 10^6^/mL).
Enhanced Monocyte recovery (Trial IV)	Volume of buffy coat bags increased to four bags and Ficoll Hypaque as density gradient medium	To improve the monocyte yield	Desired concentration achieved. (9 × 10^3^/μL) But was tedious and labour intensive and high turnaround time. (6–8 h)
Change in density gradient medium (Trial V)	Layering of buffy coat over Histopaque	To reduce processing time	No distinct monocyte layer formed for separation
Dilution of buffy coat with PBS (Trial VI)	Buffy coat was diluted with PBS in 1:1 ratio before layering over Hisopaque	Improve monocyte recovery and decrease turnaround time.	Successful monocyte count of 8.3 × 10^6^/mL mean of 5 attempts of preparation obtained. Selected for further studies. (TAT – 2‐3 h)
Culture Medium preparation (Trial VII and VIII)	Preparation and storage of RPMI – 1640 culture medium	Culture media for support	Standardized culture medium preparation established.
Trial IX – MMA procedure	Monocytes isolated as standardized above. Monocytes adherence to culture plate, cell suspension of sensitized red cells O Rh D positive cells and Anti‐D (IgG), following this the cell suspension is added to the culture plate followed by 24 h incubation, staining and microscopic evaluation	Establish MMA procedure	Cell lysis, Poor smear quality, over staining
Trial X – MMA procedure	Incubation time decreased to 1 h and the steps repeated	To standardize procedure by preventing excessive hemolysis leading to red cell fragmentation and hindering the visualization of phagocytosis.	DAT positive with a strength 2+ with 1 h incubation of cells and corresponding antisera. Test sample preparation validated.
Trial XI – MMA procedure	Comparison of 1‐h and 24‐h incubation periods of sensitized cells with monocytes followed by staining	To optimize cell morphology and visualization.	24 h led to increased hemolysis, however, 1 h incubation produced better visualization due to decreased lysis.
Trial XII – Final validation with test samples	The standardized procedure was tested with serum of AIHA patients with autoantibodies. Counting 300 monocytes was done by two different personnel, both of which yielded a similar monocyte index.	To validate the final MMA procedure with test samples	Procedure validated with adequate monocyte recovery, good microscopic visualization and repeatability.

**Demographics and clinical characteristics:** The average age of the patients tested was 47 years, ranging from 23 to 72 years, consisting of two females and three male patients. The Direct antiglobulin test was positive, and Indirect antiglobulin testing showed pan‐reactive with no pattern; suggestive of AIHA with no underlying alloantibodies. Peripheral smear showed presence of spherocytes suggestive of hemolysis with elevated indirect bilirubin and Lactate dehydrogenase (LDH). The mean hemoglobin and hematocrit were 5.6 g/dL and 18.96%, respectively.

The following observations were made while optimizing the assay conditions. The final optimized trial for each step was performed in sets of five to assess repeatability and mean values are provided where applicable

- Monocyte Isolation:
  - Initial attempts at monocyte isolation using buffy coat bags faced challenges, such as compressed bags (Trial I) and bulging of polystyrene tubes without the formation of a mononuclear layer (Trial II). These issues could be attributed to the improper centrifugation parameters.
  - Thin mononuclear layers (~100 μL) and low monocyte counts (0.25 × 10^6^/mL) in Trial III indicated inadequate separation, possibly due to insufficient buffy coat volume or suboptimal centrifugation speed and duration.
- Optimizing the variables affecting monocyte isolation:
  - Using four buffy bags and optimizing the centrifugation speed to 600  *g* for 10 min in Trial IV significantly improved the yield of monocytes (9 × 10^6^/mL), although it was time‐consuming (Turnaround time—6‐8 h).
  - Trials V and VI aimed to reduce the processing time by using histopaque with and without dilution. The best yield (8.3 × 10^6^/mL) was obtained by diluting blood from buffy coat bags with PBS (1:1) before centrifugation at 400 *g* without the brake. Following this method, the TAT for monocyte isolation was reduced to 2–3 h.
- Impact of Density Gradient Media and Centrifugation Parameters:
  - Density gradient centrifugation with Ficol Hypaque in Trial IV provided better mononuclear layer separation, highlighting the importance of the correct density gradient selection and gentle layering.
- Monocyte Quality Control (QC) and Optimization:
  - A monocyte count was performed at the end of each step of isolation to choose the best possible combination, and a count of 8.3 × 10^6^/mL was achieved in Trial VI.
- Incubation Time and Microscopic Observation:
  - A comparison of 1‐h and 24‐h incubations (Trial XI) showed that shorter incubation times resulted in better microscopic images with minimal lysis. The final optimization step (Trial XII) confirmed that a 1‐h incubation yielded consistent results under the microscope. The twenty‐four‐hour incubation period showed higher disintegration of cells leading to fragmentation and interference with reading of adherence and phagocytosis. This was improved with a 1 h incubation period optimizing cell morphology and visualization.

A pictorial representation of the final optimized steps of the monocyte monolayer assay is shown in Figure 1. Four buffy coat units derived after component separation of whole blood units were pooled, and 25 mL of buffy coat and 25 mL of phosphate‐buffered solution were added to a 50 mL centrifuge tube. To a fresh 15 mL centrifuge tube, 4 mL of density gradient medium, an equal volume of diluted sample was added, followed by centrifugation at 600 *g* for 15 min to separate the mononuclear layer. The extracted mononuclear layer was washed using phosphate‐buffered saline at 400 *g* for 10 min to ensure purity and remove any contaminants. After removing the wash supernatant, the final monocyte layer was mixed with the RPMI 1640 medium. The monocyte concentration was maintained within the target range of 3–6 × 10^6^ cells/mL. A fresh 8‐well culture plate was taken and labeled appropriately as the positive control, negative control, and donor unit identification numbers. 200 μL of mononuclear cells were added to each well of a culture plate and incubated at 37°C for 1 h. 200 μL of the test sample was added to each well and incubated for 1 h at 37°C. The supernatant was aspirated by careful pipetting, and the chamber was carefully removed according to the manufacturer's instructions. The chamber was rinsed with saline three times by careful and slow pipetting. Leishman stain and buffer were added consecutively, each with a two‐ and 10‐min incubation time. Finally, the plate was washed with distilled water and air‐dried before visualizing under a microscope. The pH was verified after monocyte preparation and before adding to the culture plate. An optimal pH of 7.3 was maintained throughout the test procedure. Table 3 summarizes the variables affecting the monocyte monolayer assay in our setup with final optimized steps.

**FIGURE 1:**
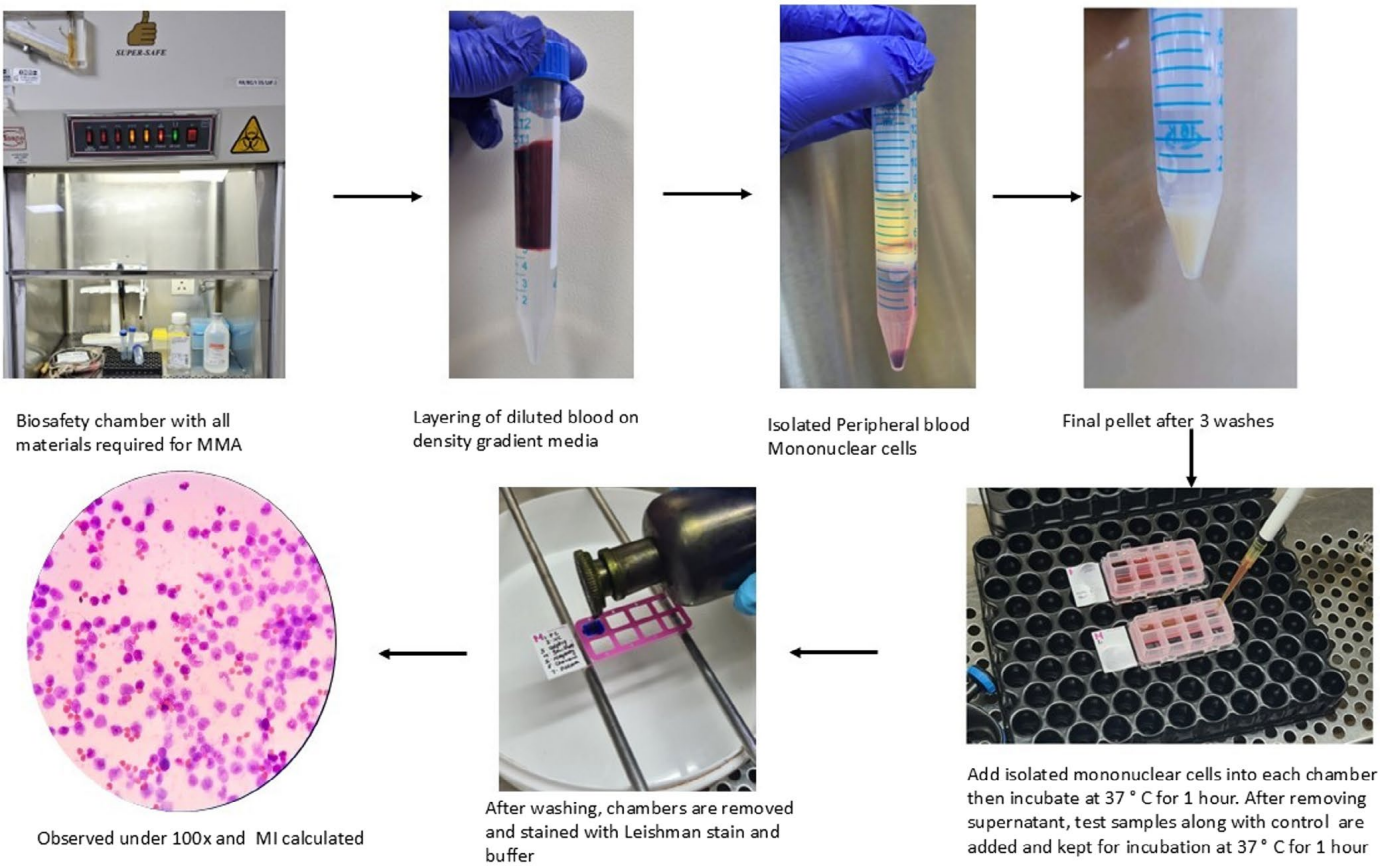
Finalized steps of monocyte monolayer assay.

**TABLE 3:** Observed key variables affecting the assay and its optimized values.

Key variables observed	Optimized value
Source of monocytes	Allogenic, 4 Buffy Coat bags
Centrifugation speed and duration (first Spin)	600 *g* for 15 min
Anticoagulant	Citrate
Diluting agent used to suspend buffy coat layer following first spin and the ratio	Phosphate Buffer at 1:1 ratio
Preferred density gradient Media	Histopaque
Centrifugation speed and duration (second Spin)	400 *g* for 10 min without brake
Incubation time and temperature for monolayer preparation and testing.	37°C for 60 min
Optimal pH	7.3
Staining	Leishman Stain
Counting for Monocyte Index	300 monocytes in each well

## 4 Discussion

Although the Monocyte Monolayer Assay (MMA) has been established for some time and offers valuable insight into its relevance to characterize the clinical significance of antibodies to red cell antigens, it remains underutilized in routine practice compared to conventional serological methods such as the indirect antiglobulin test (IAT). This limited adoption is primarily due to the assay's technical complexity, time‐intensive nature, and requirement for specialized expertise. We observed considerable variability in the protocols reported in the literature for each step of cellular assays. The most critical element of the assay was the isolation of monocytes, and there was wide variation in obtaining the source as per the literature. Autologous monocytes are preferred to ascertain the clinical significance of the red cell antibodies; however, the test is performed in reference laboratories, testing with autologous monocytes is not always feasible. Allogenic monocytes are more commonly used than autologous monocytes, and their source is peripheral blood with varying volumes of 2–50 mL [17]. Buffy coat‐derived monocytes were also studied along with cryopreservation, while retaining their functional properties for up to 6 months [16]. The resulting monocytes were plated on a tissue culture plate or glass slide and incubated at 37°C with or without carbon dioxide. Phagocytosis was observed using a light or phase‐contrast microscope.

In the current assay method, we utilized an allogenic source of monocytes, using pooled buffy coats from healthy blood donors, to enhance monocyte efficacy and decrease inter donor variability [17]. As buffy coat units are generally discarded after separation of platelet concentrates, they can be utilized for MMA without drawing an additional sample from the donor. According to the literature, citrate anticoagulants are more effective than EDTA for monocyte isolation [17]. We opted for citrate as the anticoagulant because it is present in the primary blood bag (citrate phosphate dextrose) used for collection. The buffy coat were stored at room temperature and were used 24 h after collection, following the completion of the screening tests. Adhesion of RBCs to monocytes followed by phagocytosis are the sequential stages of Fc γ receptor mediated phagocytosis [18]. This in vitro assay is a fixed‐point alternative for a continuous in vivo process, and so if only phagocytosis is assessed, an antibody that produces strong FcγR adherence but has not yet completed engulfment would be scored as falsely negative. In this study we optimized the procedure for identification of best matched units for AIHA patients and hence, we considered both adherence and phagocytosis for the calculation of monocyte index to increase the sensitivity. The overall turnaround time to perform the assay from monocyte isolation to assessing phagocytosis was approximately 8–12 h.

It has been stated that a carbon dioxide environment alone or in combination with a buffer is essential for maintaining of pH in MMA [17, 19]. However, the pH could be better maintained in the current protocol without using 5% CO_2_ during 37°C incubation of monocytes probably because of the use of CPD as an anticoagulant during the separation of monocytes, use of RPMI medium and shorter incubation periods. A 24‐h incubation of monocytes with antibody‐coated red cells produced more hemolysis and poorer microscopic morphology in our protocol, reducing the incubation to 1 h gave consistent monolayer quality with minimal lysis across repeat trials. MMA in pre‐transfusion testing was described by El‐sayed et al. where MMA was performed in conjunction with routine antibody detection using gel technique [14]. They also addressed that the 8‐well chamber‐slides were better than the 24‐well culture plates, as the latter showed a lot of un‐phagocytosed RBCs in the background. Also, Leishman staining was better than Giemsa staining with better and clearer differentiation between the RBCs, monocytes and phagocytic vacuoles. The current study also used 8‐well culture plates with Leishman stain which helped better visualize both adhesion and phagocytosis. The present study replicated the use of MMA for patients with autoantibodies but not alloantibodies to red cell antigens [14].

Noumsi et al. highlighted the use of a two‐stage complement sensitization technique, with specific incubation protocols and staining using Wright–Giemsa, which validated MMA's utility in safely transfusing antigen‐positive blood into alloimmunized patients [9, 10]. Cruz et al. utilized gelatin‐coated wells and Leishman staining to analyze monocyte activity under microscopy and provided evidence supporting MMA in defining the clinical relevance of autoantibodies, [20] while Kipkeu et al. explored the cryopreservation of monocytes from buffy coats, showing that post‐thaw monocyte recovery and membrane integrity remained intact [16]. Branch et al. emphasized the importance of timing in conducting MMA, noting that the test should be performed prior to each transfusion, especially in serologically incompatible red blood cell cases [21]. Sandhu et al. illustrated that the use of plasma instead of serum improved interpretability in the presence of complement‐activating antibodies (e.g., anti‐Vel, anti‐Jka), which previously led to excessive rosette formation, complicating phagocytosis assessments [22]. Lipman et al. described challenges in conducting MMA in patients with Sickle Cell Disease. They recommended the use of ammonium chloride for autologous red cell lysis to reduce contamination during monocyte isolation from autologous cells. Thus, highlighting the significance of adaptation of the protocol in specific patient populations and based on the type of antibody under evaluation [23].

In this study, standardization of the Monocyte Monolayer Assay for patients with autoantibodies following multiple trials led to the development of a reproducible in‐house protocol. Careful evaluation is essential for standardizing cellular assays in the laboratory, as they significantly influence assay reproducibility, reliability, and the overall interpretability of results. The steps of the procedure may be reproducible to alloantibodies; however, a careful optimization of the procedure is essential before it is generalized as it was not evaluated in the current study.

Limitation: The procedure is not compared against any other invitro cellular assays like ADCC due to non‐availability of facility and funds.

## Author Contributions

S.S. conceptualized and designed the research, supervised the research, wrote the first draft, and reviewed and edited the manuscript. K.B. performed the research, collected and analyzed the data, wrote the paper, and designed the research study. D.C. designed the study, analyzed the data, wrote the paper, and reviewed and edited the manuscript. G.M. supervised and reviewed the study protocol. K.I. performed the experiments.

## Funding

This work was supported by Terumo Penpol Private Limited. The funder had no role in the design, data collection, data analysis, or reporting of the study.

## Ethics Statement

The study protocol was reviewed and approved by the Institutional Ethics Committee of Kasturba Medical College and Kasturba Hospital Institutional Ethics Committee (approval number 471/2022). Written informed consent was obtained from the study participants in accordance with the protocol.

## Conflicts of Interest

The authors declare no conflicts of interest.

## Data Availability

The authors agree to make the data and materials supporting the results or analyses presented in their paper available upon reasonable request.
